# Supplementary material for: Statistical parametrization of cell cytoskeleton reveals lung cancer cytoskeletal phenotype with partial EMT signature
Source: Commun Biol. 2022 May 2;5:407. doi: 10.1038/s42003-022-03358-0 (PMC9061773; doi:10.1038/s42003-022-03358-0)
Supplement: Supplementary file 2 — Supplementary Information [file 42003_2022_3358_MOESM2_ESM.pdf]

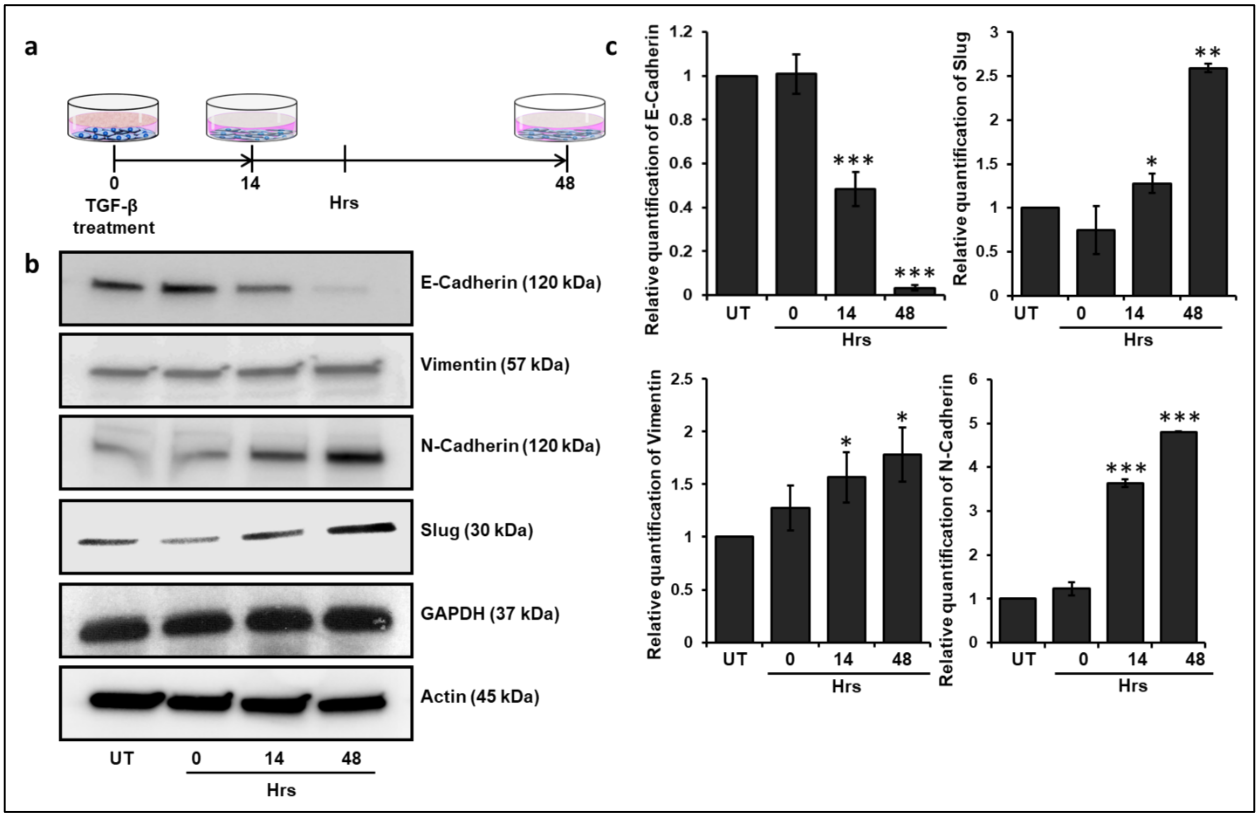

Supplementary Fig.1 | Expression of EMT EMT marker proteins. a, Cartoon Schematic of the TGFβ1 treatment experiment. **b,** Intensity bands of specific EMT marker proteins at different time points done by western blot analysis (actin and GAPDH used as reference). **c,** Densitometric analyses showing the relative amounts of EMT marker proteins shown in **b**. All data are presented as mean ± SEM. $^{*}p < 0.05$, $^{**}p < 0.01$, $^{***}p < 0.001$.

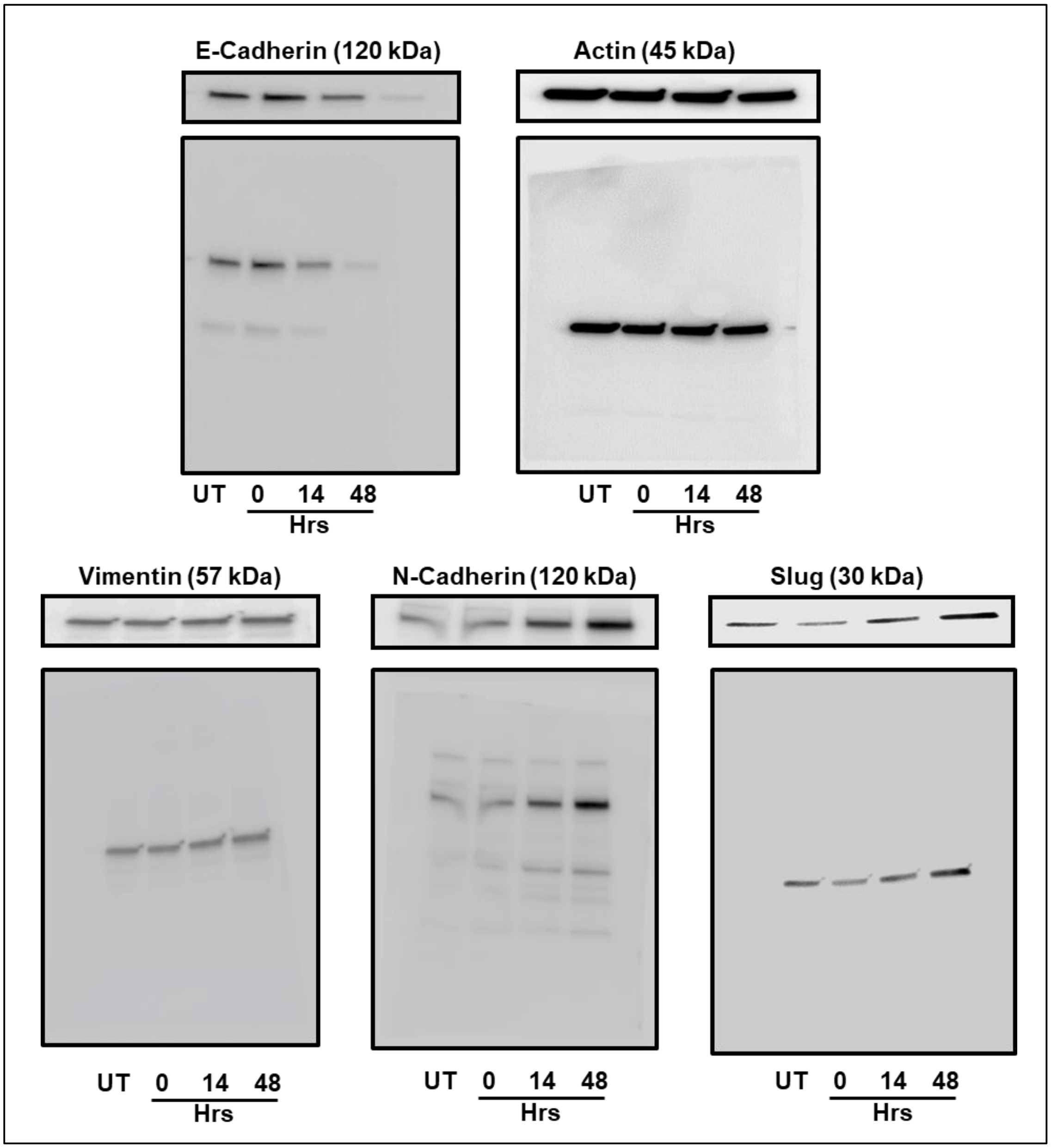

Supplementary Fig. 2 | Uncropped and unprocessed western blot images for E-Cadherin, Actin, Vimentin, N-Cadherin and Slug.

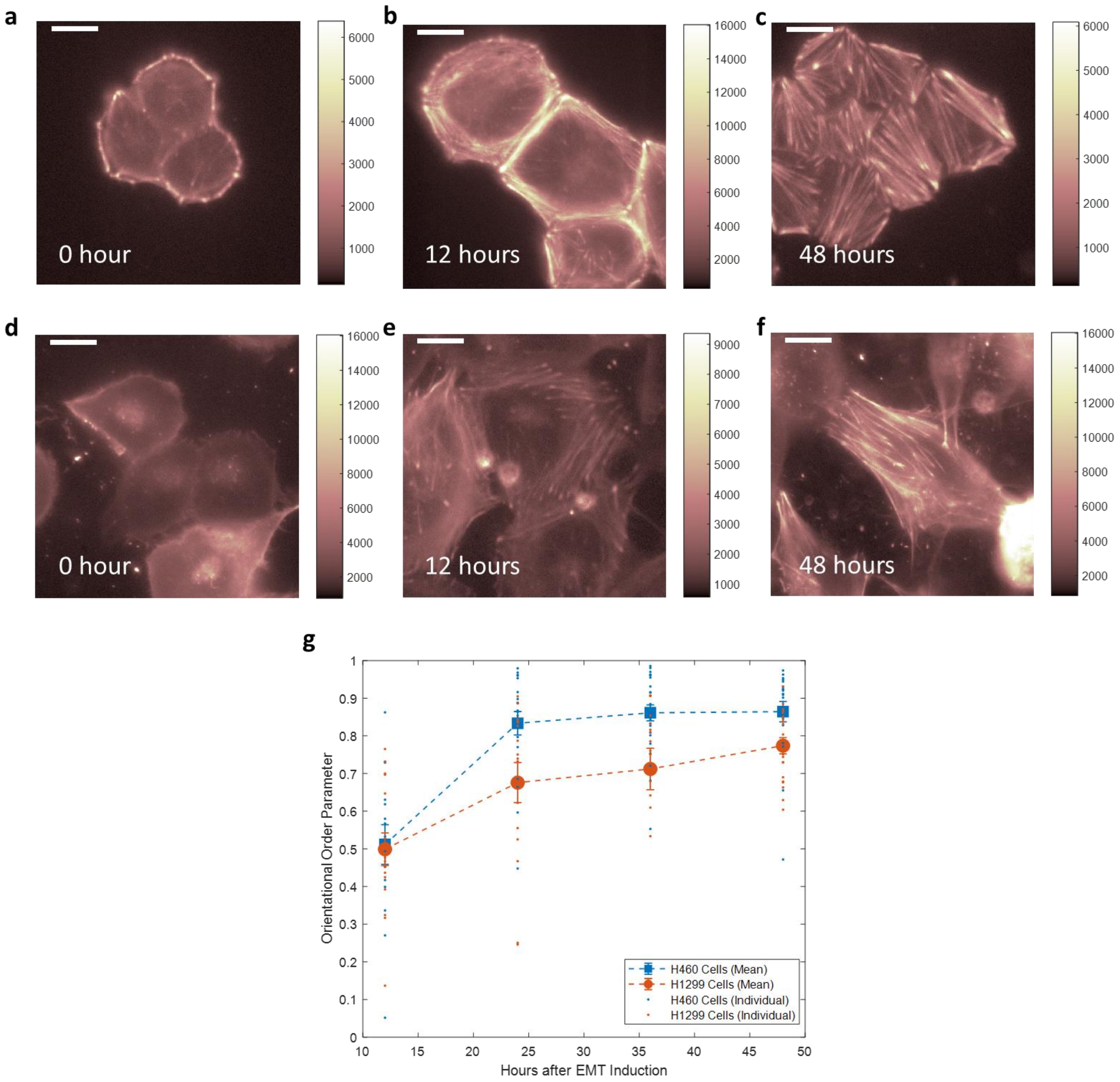

Supplementary Fig. 3 | H460 and H1299 cell lines undergoing EMT stained with SiR-actin. a-c, H460 cells at 0, 12 and 48 hours after addition of TGFβ1 respectively. **d-f,** H1299 cells at 0, 12 and 48 hours after addition of TGFβ1 respectively. **g,** Plots of individual and mean OOP values vs time of TGFβ1 treatment for H460 (blue) and H1299 (red) respectively. Error bars correspond to standard error at respective time-points. H460: n=15 (for 12 hours), n=20 (for 24hours), n=25 (for 36hours) and n=20 (for 48hours). H1299: n=16 (for 12hours), n=16 (for 24hours), n=15 (for 36hours) and n=20 (for 48hours). Scale bar: 16μm.

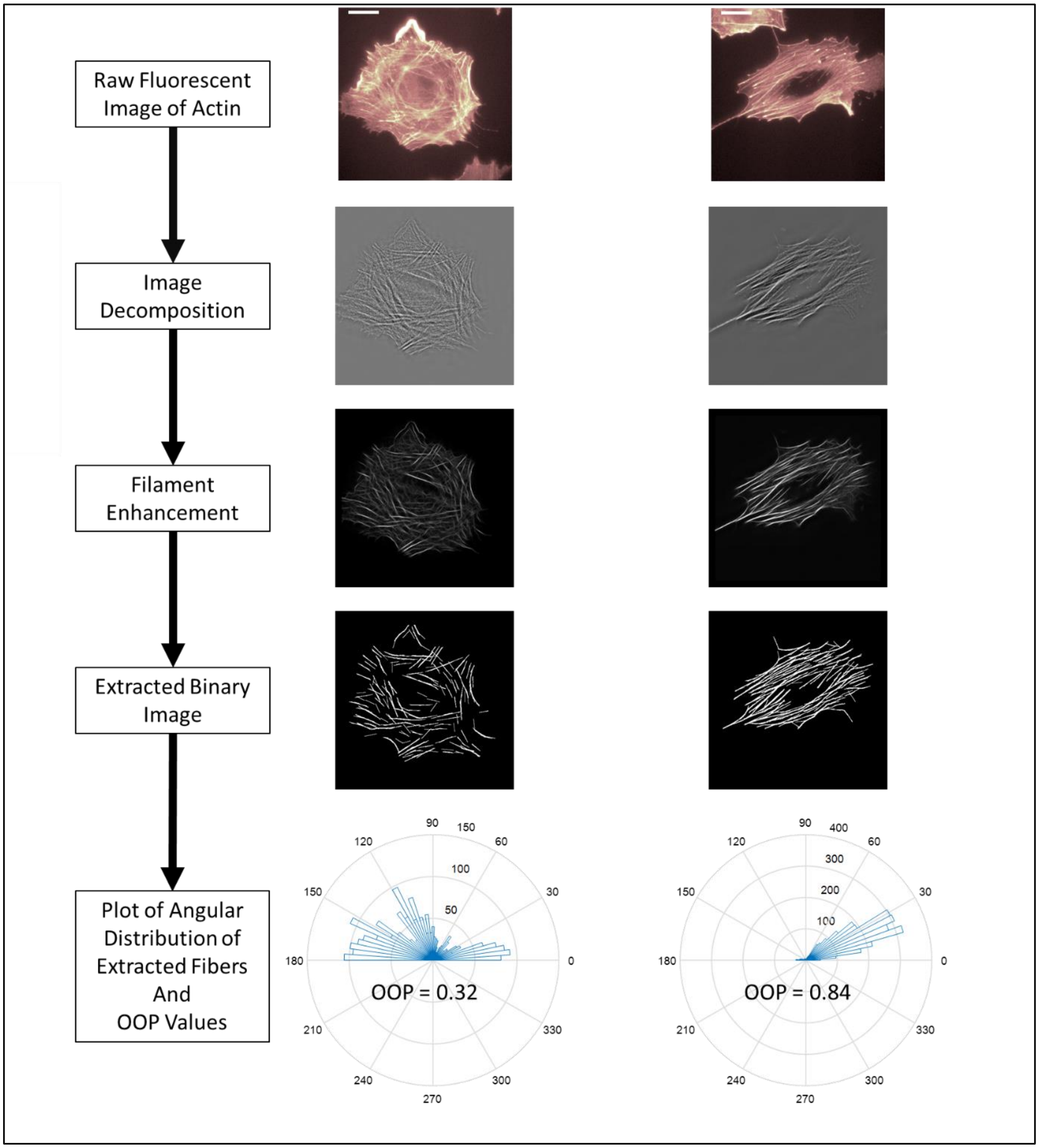

Supplementary Fig. 4 | Flowchart for image analysis and quantification. Scale bar: 16μm.

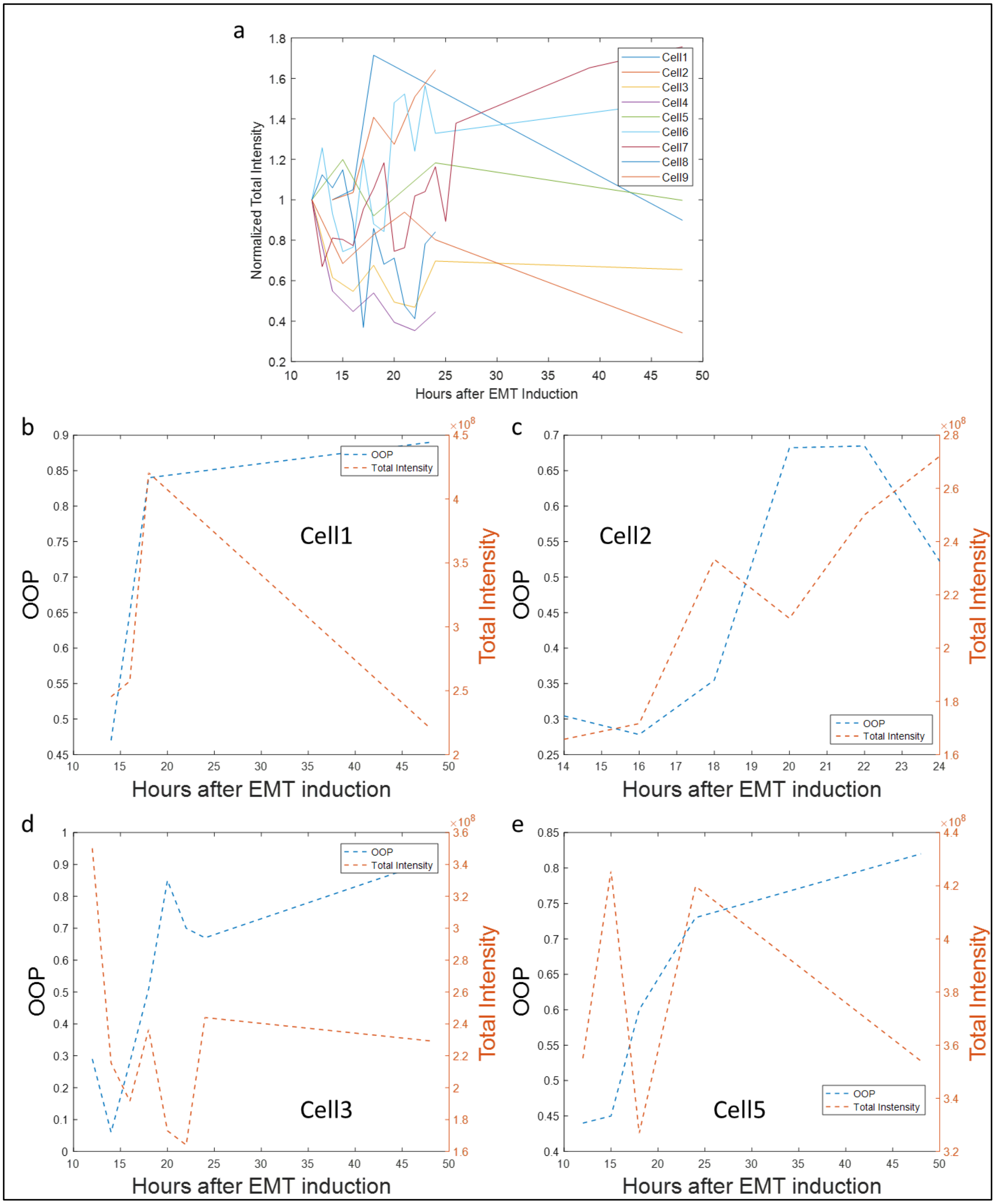

Supplementary Fig. 5 | Variation of total intensity of live cells undergoing EMT. a, Plot of normalized total intensity of fluorescent actin images vs. hours of TGFβ1 treatment demonstrating random variation in intensities. The intensity series for each cell was normalized with respect to the intensity of the first image of that cell. **b-e,** Plots of OOP and total intensities of Cell 1, Cell 2, Cell 3 and Cell 4 respectively showing four cells with overall increasing OOPs can have different intensity trajectories.

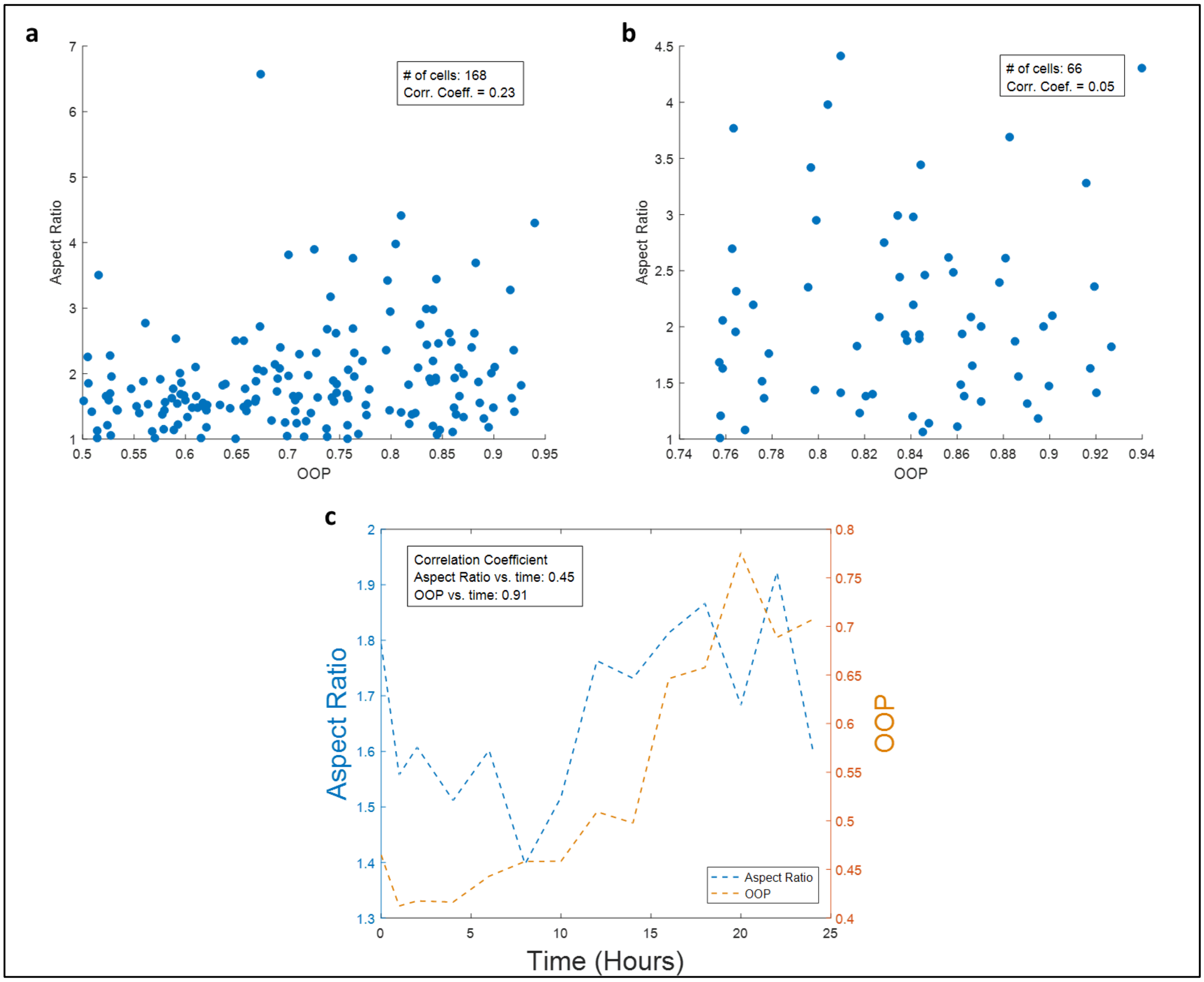

Supplementary Fig. 6 | Distribution of Aspect Ratio of cells with respect to their OOPs. a, Plot of aspect ratio of cells (n = 168) vs. their corresponding OOPs (OOP > 0.5) demonstrating poor correlation between aspect ratio and OOP (Correlation Coefficient = 0.23). **b,** Plot of aspect ratio of cells (n = 66) vs. their corresponding OOPs (OOP > 0.75) demonstrating correlation between aspect ratio and OOP getting worse for cells with higher OOP (Correlation Coefficient = 0.05). **c,** Plot of mean aspect ratio (Correlation Coefficient: 0.45) and OOP (Correlation Coefficient: 0.91) of cells (n = 297) against hours of TGFβ1 treatment.

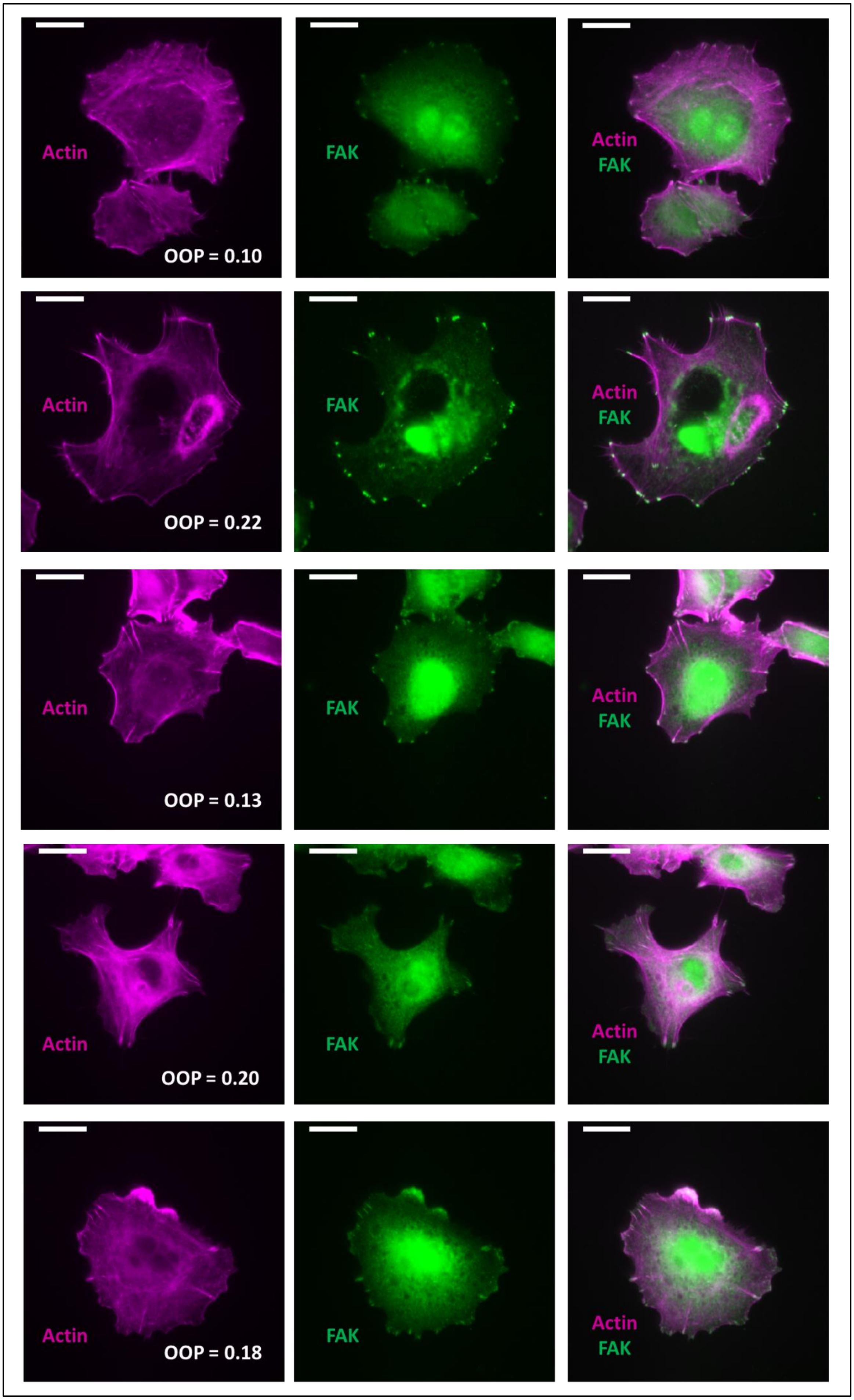
Actin
OOP = 0.10
FAK
Actin
FAK
Actin
OOP = 0.22
FAK
Actin
FAK
Actin
OOP = 0.13
FAK
Actin
FAK
Actin
OOP = 0.20
FAK
Actin
FAK
Actin
OOP = 0.18
FAK
Actin
FAK

Supplementary Fig. 7 | Focal Adhesion Kinase (FAK) pattern of multiple low OOP cells. Fluorescent image of cell with disoriented actin stress fibers (magenta, left panels), fluorescent image of FAK (green, middle panels) of the same cell shown in left panels FAK spots near the cell-edge. Overlay image (right panels) of actin (magenta) and FAK (green) of the cells showing stress fibers with zero or one FAK capping. Scale bar: 16μm.

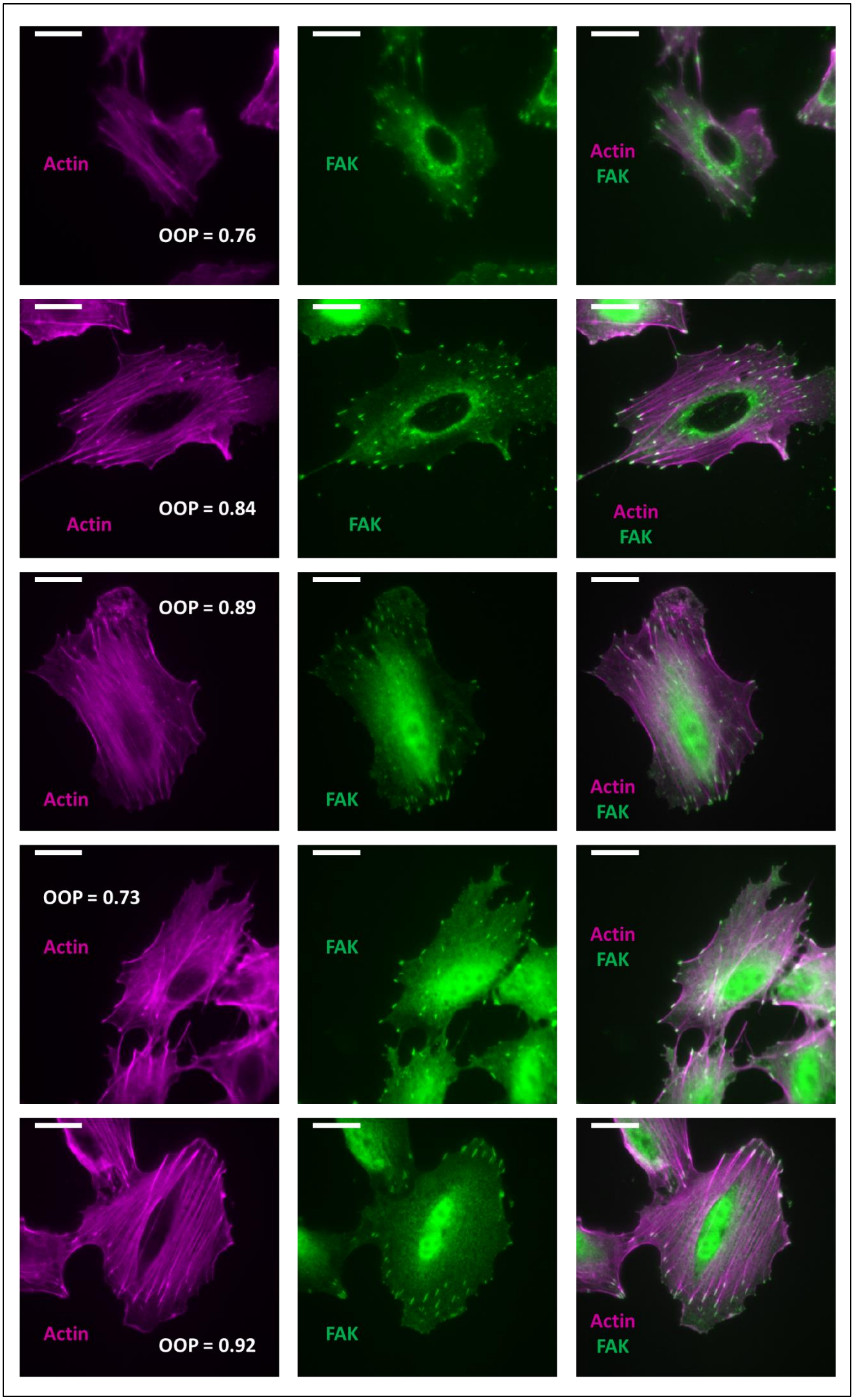

Actin
OOP = 0.76
FAK
Actin
FAK
OOP = 0.84
Actin
FAK
Actin
FAK
OOP = 0.89
Actin
FAK
Actin
FAK
OOP = 0.73
Actin
FAK
Actin
FAK
Actin
OOP = 0.92
FAK
Actin
FAK

Supplementary Fig. 8 | Focal Adhesion Kinase (FAK) pattern of multiple high OOP cells. Fluorescent image of cell with disoriented actin stress fibers (magenta, left panels), fluorescent image of FAK (green, middle panels) of the same cell shown in left panels FAK spots throughout the cell. Overlay image (right panels) of actin (magenta) and FAK (green) of the cells showing stress fibers with one or two FAK capping. Scale bar: 16μm.

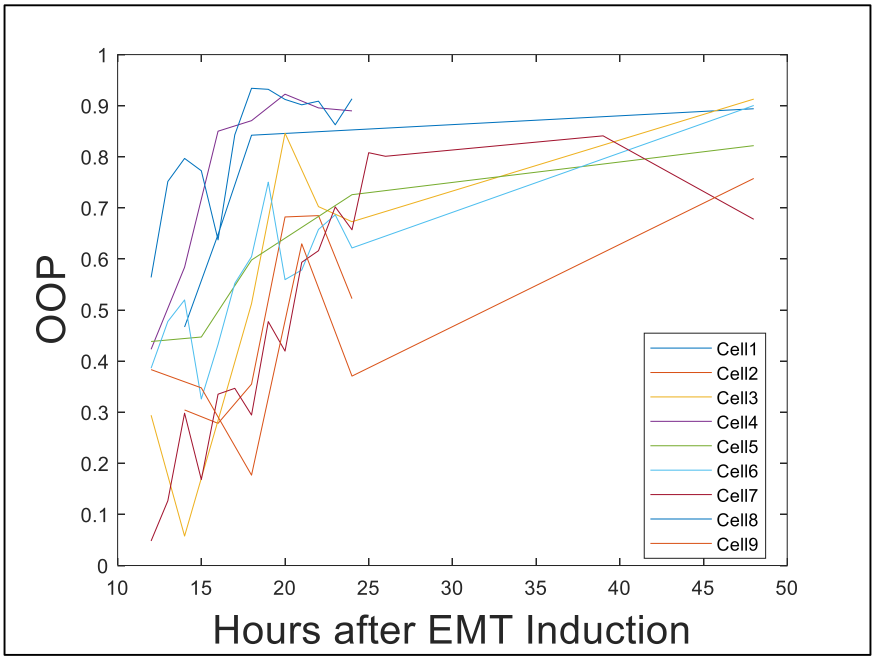

Supplementary Fig. 9 | Plot of multiple single cell OOP trajectories with time of EMT induction.

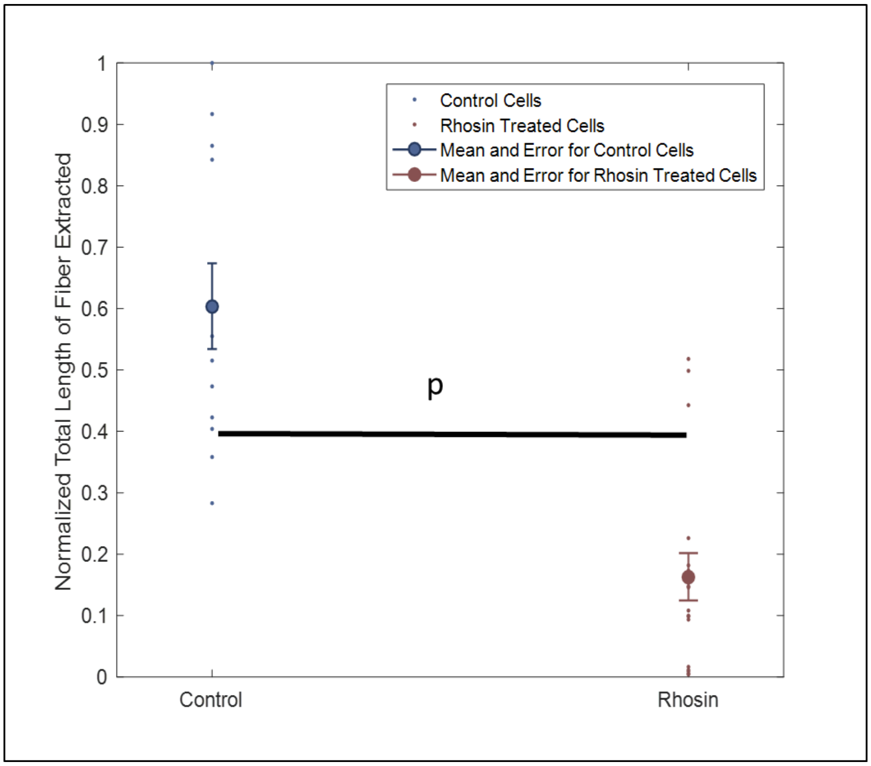

Supplementary Fig. 10 | Comparison of length of actn fibers extracted from fluorescent images. Every length is normalized with respect to the length of the longest extracted fiber. Control: n=12. Rhosin: n=18. $p = 7.97 \times 10^{-7}$.

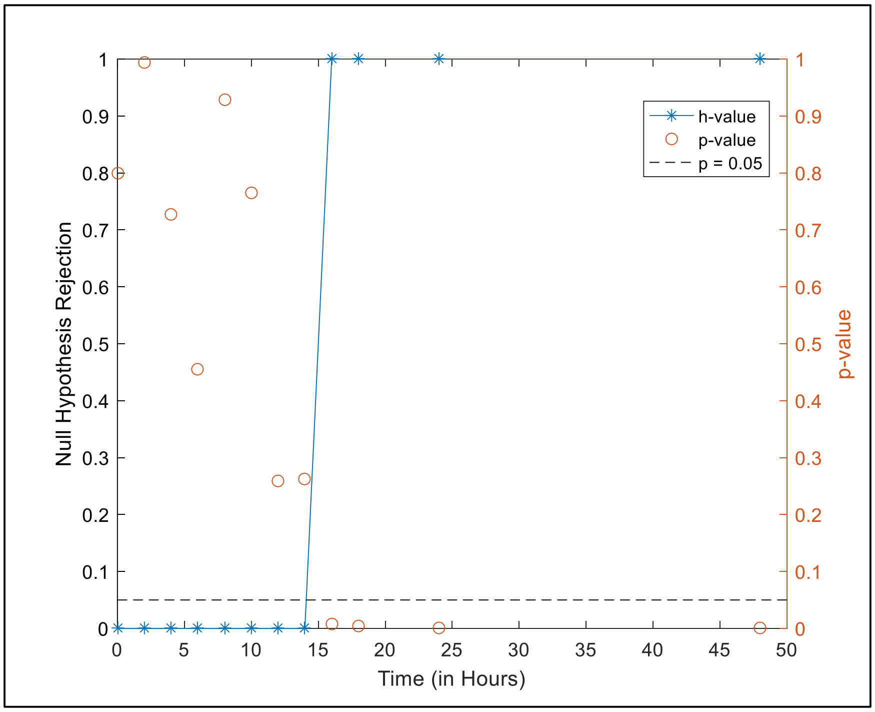

Supplementary Fig. 11 | Statistical comparison of cell populations with and without Tankyrase treatment showing significant difference in OOP from 16-48 hours. We have conducted the T-test on the cell populations with and without Tankyrase at the same time points. The h-value (left hand Y-axis) or the binary rejection of null hypothesis (the two data sets belong to independent normal distributions with the same mean) shows that the hypothesis is rejected for time-points 16 hours and later, i.e., after 16 hours, drug treatment creates a significant difference on the OOP values. The p-value (right hand Y-axis) clearly demonstrates that the p-value is larger than the cut-off 0.05 for 0-14 hour time-points indicating that the validity of the null hypothesis for those time-points can occur due to randomness and as such is not significant. p-values on or after 16 hours are all below 0.05 indicating the rejection of the null hypothesis is significant.

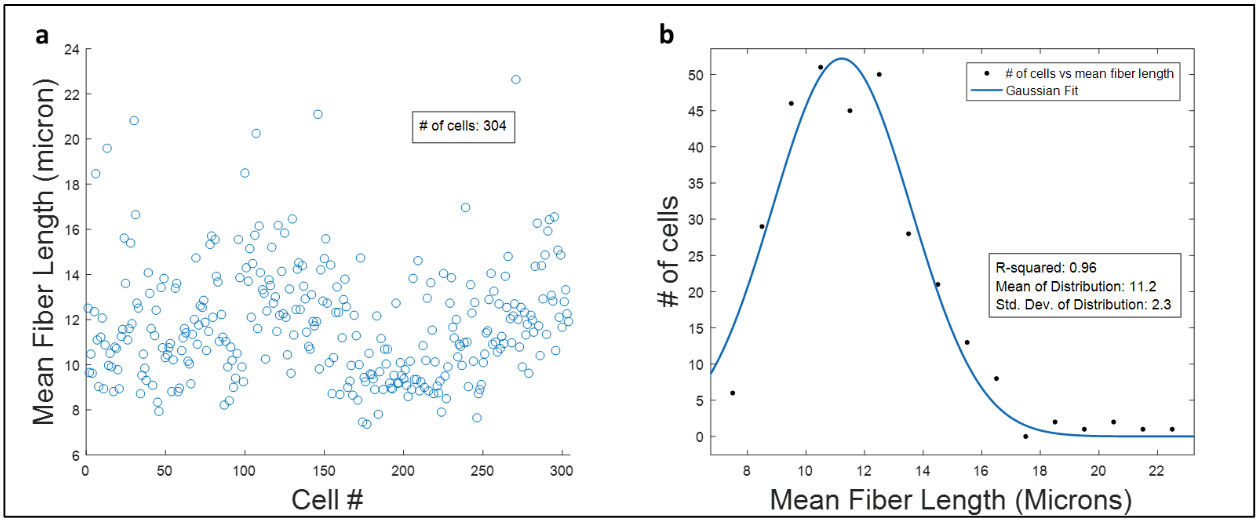

Supplementary Fig. 12 | Variation of mean fiber lengths of A549 cells. a, Plot of mean fiber length of 304 cells demonstrating most cells have a mean fiber length between 8-14 μm. **b,** Gaussian fit of mean fiber lengths with a mean 11.2 μm and standard deviation 2.3 μm.
