# Supplementary material for: Statistical parametrization of cell cytoskeleton reveals lung cancer cytoskeletal phenotype with partial EMT signature
Source: Commun Biol. 2022 May 2;5:407. doi: 10.1038/s42003-022-03358-0 (PMC9061773; doi:10.1038/s42003-022-03358-0)
Supplement: Supplementary file 3 — Description of Additional Supplementary Files [file 42003_2022_3358_MOESM3_ESM.pdf]

Description of Additional Supplementary Files

File name: Supplementary Video 1
Description: **Tracking phenotypic transition of a single cell.** (Left panel) Fluorescence images of a single A549 cell stained with SiR-Actin at the specified time-points after addition of TGFβ1 addition. (Right panel) OOP values extracted from the corresponding fluorescence image in the left panel.

File name: Supplementary Data 1
Description: Individual data points used to generate the figures in the main manuscript.
