# Supplementary material for: Statistical parametrization of cell cytoskeleton reveals lung cancer cytoskeletal phenotype with partial EMT signature
Source: Commun Biol. 2022 May 2;5:407. doi: 10.1038/s42003-022-03358-0 (PMC9061773; doi:10.1038/s42003-022-03358-0)
Supplement: Supplementary file 6 — Reporting Summary [file 42003_2022_3358_MOESM6_ESM.pdf]

nature research

Corresponding author(s): Shimon Weiss

Last updated by author(s): Mar 22, 2022

Reporting Summary

Nature Research wishes to improve the reproducibility of the work that we publish. This form provides structure for consistency and transparency in reporting. For further information on Nature Research policies, see our Editorial Policies and the Editorial Policy Checklist.

Statistics

For all statistical analyses, confirm that the following items are present in the figure legend, table legend, main text, or Methods section.

n/a	Confirmed	
☐	☒	The exact sample size (n) for each experimental group/condition, given as a discrete number and unit of measurement
☐	☒	A statement on whether measurements were taken from distinct samples or whether the same sample was measured repeatedly
☐	☒	The statistical test(s) used AND whether they are one- or two-sided<br>*Only common tests should be described solely by name; describe more complex techniques in the Methods section.*
☒	☐	A description of all covariates tested
☐	☒	A description of any assumptions or corrections, such as tests of normality and adjustment for multiple comparisons
☐	☒	A full description of the statistical parameters including central tendency (e.g. means) or other basic estimates (e.g. regression coefficient) AND variation (e.g. standard deviation) or associated estimates of uncertainty (e.g. confidence intervals)
☐	☒	For null hypothesis testing, the test statistic (e.g. F, t, r) with confidence intervals, effect sizes, degrees of freedom and P value noted<br>*Give P values as exact values whenever suitable.*
☒	☐	For Bayesian analysis, information on the choice of priors and Markov chain Monte Carlo settings
☒	☐	For hierarchical and complex designs, identification of the appropriate level for tests and full reporting of outcomes
☒	☐	Estimates of effect sizes (e.g. Cohen's d, Pearson's r), indicating how they were calculated

Our web collection on statistics for biologists contains articles on many of the points above.

Software and code

Policy information about availability of computer code

Data collection	Andor SOLIS for Imaging software was used for fluorescent microscopy. Nanowizard 4A SPM Desktop Software was used for AFM force curve measurements.
Data analysis	All AFM force curves were analyzed using JPKSPM Data Processing Software. MCALab Signal and Image Decomposition and Inpainting library was used for part of the image extraction pipeline. All custom codes used for image cropping, image analysis, data visualization and OOP calculation are published as a GitHub repository (https://github.com/arkaprabha/Statistical-Parametrization-of-Cell-Cytoskeleton-SPOCC).

For manuscripts utilizing custom algorithms or software that are central to the research but not yet described in published literature, software must be made available to editors and reviewers. We strongly encourage code deposition in a community repository (e.g. GitHub). See the Nature Research guidelines for submitting code & software for further information.

Data

Policy information about availability of data

All manuscripts must include a data availability statement. This statement should provide the following information, where applicable:

- Accession codes, unique identifiers, or web links for publicly available datasets
- A list of figures that have associated raw data
- A description of any restrictions on data availability

The data that support the findings of this study are available from the corresponding author upon request. All data underlying plots or graphs in the figures in the main manuscript have been added as supplementary material.

April 2020

Field-specific reporting

Please select the one below that is the best fit for your research. If you are not sure, read the appropriate sections before making your selection.

☒ Life sciences ☐ Behavioural & social sciences ☐ Ecological, evolutionary & environmental sciences

For a reference copy of the document with all sections, see nature.com/documents/nr-reporting-summary-flat.pdf

Life sciences study design

All studies must disclose on these points even when the disclosure is negative.

Sample size	Sample size was not chosen based on any statistical analysis. We imaged enough cells that represent the overall characteristic of the whole population. Thus, the sample size is different for different experiments. For most of the experiments, the sample size varies between 9 and 25.
Data exclusions	Cells that looked unhealthy or damaged were not analyzed. If any cell demonstrated completely different extracted fiber pattern from the fluorescent image, then they were re-analyzed to check for errors and rejected. For live cell trajectories, if the cell being tracked could not be confidently identified amongst its neighbors, then that data was rejected.
Replication	Data represented in Fig. 1g is a combination of three replicate measurements. They all demonstrated low OOP at earlier time points and high OOP at later time points. The nine live cell trajectories shown in Fig. 3 were taken from experiments done on six different days. Data represented in Fig. 4g was replicated in another experiment. AFM experiment was carried out only once.
Randomization	N/A
Blinding	N/A

Reporting for specific materials, systems and methods

We require information from authors about some types of materials, experimental systems and methods used in many studies. Here, indicate whether each material, system or method listed is relevant to your study. If you are not sure if a list item applies to your research, read the appropriate section before selecting a response.

Materials & experimental systems

n/a	Involved in the study	
☐	☒	Antibodies
☐	☒	Eukaryotic cell lines
☒	☐	Palaeontology and archaeology
☒	☐	Animals and other organisms
☒	☐	Human research participants
☒	☐	Clinical data
☒	☐	Dual use research of concern

Methods

n/a	Involved in the study	
☒	☐	ChIP-seq
☒	☐	Flow cytometry
☒	☐	MRI-based neuroimaging

Antibodies

Antibodies used	1. GAPDH, Advanced Immuno Chemical Inc, 2-RGM2<br>2. β-Actin, Cell Signaling Technology, 12262<br>3. E-Cadherin, BD Transduction Laboratories, 610182<br>4. N-Cadherin, BD Transduction Laboratories, 610920<br>5. Slug, Cell Signaling Technology, 9585<br>6. FAK(D1), Santa Cruz Biotechnology, sc-271126<br>7. Goat anti-mouse IgG, LI-COR Biosciences, 926-32210<br>8. Goat anti-rabbit IgG, LI-COR Biosciences, 926-68071<br>9. Donkey anti-mouse IgG, Jackson ImmunoResearch Laboratories Inc., 715-546-151
Validation	All primary antibodies used were validated by western blots by the manufacturer for human cell lines.

Eukaryotic cell lines

Policy information about cell lines

Cell line source(s)	All cell lines were purchased from ATCC.
Authentication	N/A

Mycoplasma contamination	N/A
Commonly misidentified lines<br>(See ICLAC register)	N/A
